# Structural and functional consequences of age-related isomerization in α-crystallins

**DOI:** 10.1074/jbc.RA118.007052

**Published:** 2019-02-25

**Authors:** Yana A. Lyon, Miranda P. Collier, Dylan L. Riggs, Matteo T. Degiacomi, Justin L. P. Benesch, Ryan R. Julian

**Affiliations:** From the ‡ Department of Chemistry, University of California, Riverside, Riverside, California 92521,; the § Department of Chemistry, Physical and Theoretical Chemistry Laboratory, University of Oxford, South Parks Road, Oxford OX1 3QZ, United Kingdom, and; the ¶ Department of Chemistry, Durham University, South Road, Durham DH1 3LE, United Kingdom

**Keywords:** aging, mass spectrometry (MS), radical, chaperone, protein structure, protein self-assembly, protein phosphorylation, protein chemical modification, molecular dynamics, epimer

## Abstract

Long-lived proteins are subject to spontaneous degradation and may accumulate a range of modifications over time, including subtle alterations such as side-chain isomerization. Recently, tandem MS has enabled identification and characterization of such peptide isomers, including those differing only in chirality. However, the structural and functional consequences of these perturbations remain largely unexplored. Here, we examined the impact of isomerization of aspartic acid or epimerization of serine at four sites mapping to crucial oligomeric interfaces in human αA- and αB-crystallin, the most abundant chaperone proteins in the eye lens. To characterize the effect of isomerization on quaternary assembly, we utilized synthetic peptide mimics, enzyme assays, molecular dynamics calculations, and native MS experiments. The oligomerization of recombinant forms of αA- and αB-crystallin that mimic isomerized residues deviated from native behavior in all cases. Isomerization also perturbs recognition of peptide substrates, either enhancing or inhibiting kinase activity. Specifically, epimerization of serine (αASer-162) dramatically weakened inter-subunit binding. Furthermore, phosphorylation of αBSer-59, known to play an important regulatory role in oligomerization, was severely inhibited by serine epimerization and altered by isomerization of nearby αBAsp-62. Similarly, isomerization of αBAsp-109 disrupted a vital salt bridge with αBArg-120, a contact that when broken has previously been shown to yield aberrant oligomerization and aggregation in several disease-associated variants. Our results illustrate how isomerization of amino acid residues, which may seem to be only a minor structural perturbation, can disrupt native structural interactions with profound consequences for protein assembly and activity.

## Introduction

Long-lived proteins are important but often underappreciated, with recent findings illustrating their pervasiveness within critical organs and suggesting that their chemistry and biology should not be ignored (1, 2). Longevity renders proteins susceptible to degradation via nonenzymatic, spontaneous chemical modifications, including truncation, cross-linking, oxidation, deamidation, isomerization, and epimerization (1, 3). Aspartic acid residues are most prone to isomerization (4), readily forming a succinimide ring following attack of the side chain by the peptide backbone. The succinimide is susceptible to racemization and can reopen in two ways, ultimately yielding four isomers: l-Asp, d-Asp, l-isoAsp, and d-isoAsp (5). The time scale for this process is likely dependent on many factors and will vary for each protein, but knockout experiments in mice have demonstrated that absence of the repair enzyme that targets l-isoAsp leads to fatal consequences within 4–6 weeks (6, 7).

**Figure 1.**
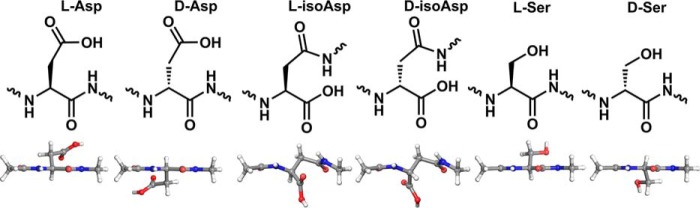
**Corresponding chemical and three-dimensional structures of the isomers and epimers examined herein.** 3D structures are shown from above, with the backbone perpendicular to the plane of the page.

Deamidation of asparagine can also yield four isomers of aspartic acid, but this process involves chemical substitution in addition to isomerization. Aspartic acid isomerization is one of the most prevalent degradation pathways of crystallin proteins in aged human lenses and is known to increase with age. Serine is also frequently found to undergo isomerization in long-lived proteins; this localized chiral inversion, known as epimerization, produces d-Ser (4, 8). Isomerization and epimerization (illustrative examples shown in Fig. 1) are difficult to detect because they do not lead to a change in mass and are consequently invisible to mass spectrometry (MS)-based methods typically employed during proteomic analyses (9, 10). As a result, they are not widely studied and are frequently overlooked, and their consequences at the molecular level are largely unknown. Among the few previously reported examples, a study of hen egg white lysozyme with an l-isoAsp substitution at Asp-101 caused a backbone deflection of nearly 90° relative to the native structure (11), and l-isoAsp-32 insertion into RNase forms a protruding U-shaped loop bent by nearly 90° instead of an α-helix (12). Isomerization can also affect physical properties such as solubility and bioactivity, with isomerization of Asp-92 in immunoglobulin γ2 (IgG2) leading to deactivation of the antigen-binding region (13).

The crystallin proteins of the eye lens, in which there is no protein turnover, are among the longest-lived proteins in the body (14). They are ideal targets for studying spontaneous degradation pathways that occur due to aging, and experiments have previously revealed that isomerization compromises water solubility and oligomerization (15–18). The most abundant crystallins in humans, αA and αB, are important molecular chaperone (19) and regulatory proteins (20). Although αA is localized almost exclusively to the eye lens (21), αB is found throughout the body (22, 23). Crystallin malfunction due to mutation or accumulation of PTMs^4^ is associated with a variety of diseases, including cataract, cardiomyopathies, motor neuropathies, and neurodegeneration (3, 24, 25).

**Figure 2.**
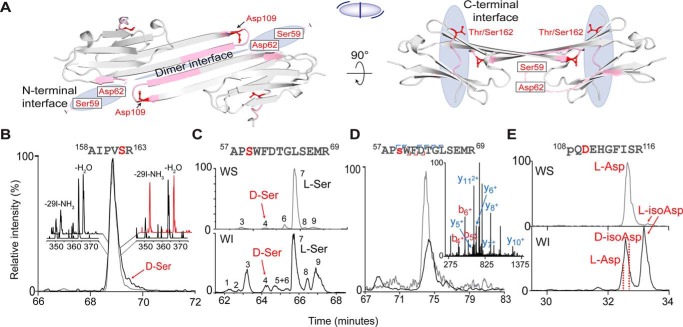
*A,* two views of the partial crystal structure of α-crystallin (αB, PDB 4M5S). The structure of αB is used for illustration purposes because αB and αA intermix freely and share high structural similarity. *Blue-shaded regions* indicate crucial oligomeric interfaces. *Pink ribbons* denote the isomer-containing peptides, with specific isomerization sites labeled in *red*. The *small cartoon in the middle* represents the assembly, with each *half-ellipse* representing a monomer, the *central line* indicating the dimer interface, and the *peripheral lines* representing bound C-terminal peptides. Extracted ion chromatograms: *B,* αA^158^AIPVSR^163^ from the WI (*black trace*) and WS (*gray trace*) fractions of the cortex. *Insets*, RDD mass spectra from the leading and trailing edge of each peak. *C,* αB, ^57^APSWFDTGLSEMR^69^ from the nucleus, revealing abundant isomerization in the WI fraction. *D,* phosphorylated ^57^AP*s*WFDTGLSEMR^69^ detected in the WS cortex (*gray trace*) and WI cortex (*black trace*), revealing far less isomerization (where *s* = phosphoserine). *Inset*, MS/MS pinpoints the site of phosphorylation to Ser-59. *E,* αB, ^108^*pQ*DEHGFISR^116^ from the cortex (where *pQ* = pyroglutamate). The abundance of l-isoAsp is much higher in the WI than WS fraction.

αA and αB are members of the small heat-shock protein family (19), with structures characterized by a highly conserved α-crystallin domain (26) flanked in both proteins by less ordered N- and C-terminal regions (Fig. 2*A*). Outside of the α-crystallin domain, αA and αB share only modest sequence homology (21). Despite this, αA and αB self- and co-assemble into large, polydisperse, and dynamic oligomers. A number of interfaces mediate this oligomerization, which begins with dimerization between pairs of ordered α-crystallin domains. The dimers then associate into oligomers via an interface between a palindromic sequence in the C-terminal region of one dimer and the α-crystallin domain of another, as well as interactions involving N-terminal regions (Fig. 2*B*) (27–29). Perturbation of the dimer interface, as occurs in the well-known R120G variant of αB (30) or mutations in the terminal regions, can lead to protein aggregation and malfunction (31). The chaperone capacity and localization of αB are also partially regulated by phosphorylation of three serines in the N-terminal region, with dysregulation leading to disease (32–36).

We recently reported 81 sites of isomerization in human αA and αB isolated from the eye lenses of aged donors (17). Here, we examine the structural consequences of isomerization at four of these sites that reside in regions critical for oligomerization, and our experiments demonstrate how key structural interactions or functionality are disrupted by isomerization. Native MS (37, 38) in conjunction with synthetic isomers, enzymatic assays, and molecular dynamics (MD) simulations were used to probe the structural consequences. Our results demonstrate that age-related isomerization of individual amino acid residues can have significant impact on the quaternary structure and function of α-crystallins, and they suggest that similar “invisible” PTMs in other long-lived proteins may have important influences on age-related diseases.

## Results and discussion

### αA and αB accumulate isomeric PTMs at their interfaces

The structures of the α-crystallin domains of αA- and αB-crystallin are very similar. Therefore, to orient where sites of interest reside in both proteins, the α-crystallin domain of αB is used as a representative model in Fig. 2*A*. In the process of oligomeric assembly, monomers first come together to form dimers. As seen in Fig. 2*A*, *left*, the dimer interface is defined by an anti-parallel β-sheet formed by the same sequence region from two different monomers. Hydrogen bonding along the intermolecular β-sheet and two complementary Arg-120–Asp-109 salt bridges stabilize the interface. Another crucial interaction occurs in a groove where the normally disordered C-terminal tail can bind (Fig. 2*A*, *right*). This interface is one of several that facilitates the assembly of dimers into larger oligomers. The disordered N-terminal tail also forms quaternary interactions favoring oligomerization in the general region shown in Fig. 2*A*, *left*, although the interaction partners are omitted for clarity. All of these regions are critical for proper crystallin assembly and function, suggesting that structural perturbations within these interfaces could have undesirable consequences.

Previous analysis identified (17) (without detailed study) four sites of isomerization within these regions. Given the potential structural impact, we now examine these sites in detail. Identification of isomerization and epimerization first requires proteolysis. Tryptic digestion of the crystallins yields three peptides that contain the four sites of interest (underlined): αA-^158^AIPVSR^163^, which lies in the C-terminal region; αB-^57^APSWFDTGLSEMR^69^, which encompasses residues involved in an N-terminal interface; and αB-^108^QDEHGFISR^116^, which lies in the α-crystallin domain at the dimer interface. Each of these peptides corresponds to one of the *pink* regions in Fig. 2*A*. The degree of isomerization or epimerization at each site was determined with RDD-MS, as described in detail previously (17), and the results are shown in Fig. 2, *B–E* and Tables S1–S4. These data were obtained from the nucleus and cortex of a 72-year-old human lens, although the results are similar to those from younger donor lenses (data not shown). The complete crystallin sequences, including all known isomerized and epimerized residues, are listed for reference in Fig. S1 along with additional details about isomer identification in Fig. S2.

**Figure 3.**
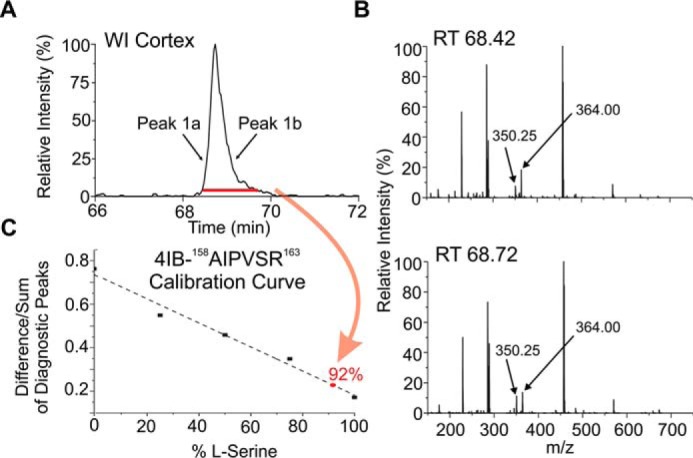
*A,* selected-ion chromatogram for 4IB-AIPVSR in the WI cortex digest of the 72-year-old lens. *B,* RDD spectra from the leading (*peak 1a*) and trailing (*peak 1b*) edges of the corresponding LC peak. *C,* calibration curve is then used to quantify the amount of d-Ser that co-elutes in the LC chromatogram. The *curve* is generated by making standard solutions that contain known amounts of both isomers and taking the difference over the sum of the two peaks that have the largest differences in the fragmentation spectra. For this peptide, the–29I–NH_3_ losses from the precursor ion and the –H_2_O loss from the precursor ion were chosen as the diagnostic peaks. The percent d-Ser/l-Ser in the digest is then determined by averaging the RDD spectra for the entire peak in *A* (indicated by the *red bar*). This value maps to the *red point* in *C*, 92% l-Ser and 8% d-Ser.

RDD-MS results for αA-^158^AIPVSR^163^ are shown in Fig. 2*B*. The ratio of the product ions at 350.25*z* and 364.00 *m*/*z* varies with elution time across the chromatographic peak for the water-insoluble (WI) fraction (compare the *red* and *black* rear mass spectra in Fig. 2*B*). In contrast, the RDD spectra for the water-soluble (WS) fraction do not vary as a function of retention time, as shown in the forward mass spectra of Fig. 2*B*. The WS spectra both match RDD data obtained from a synthetic l-Ser isomer. The changing product ion ratios for the WI fraction are meaningful because the radical in RDD is created photolytically (39) at a single, atomically precise location in both isomers. Subsequent collisional activation stimulates migration of the radical and fragmentation of the peptide. Differences in three-dimensional structure due to isomerization therefore lead to differences in radical migration, which impacts the abundance of certain fragment ions. Therefore, the varying product ion ratios for the WI fraction in Fig. 2*B* reveal isomerization of the peptide, even though this modification is not readily apparent by chromatography. Further analysis with synthetic standards confirms epimerization at ^αA^Ser-162 and enables quantification of the abundance of ^αA^d-Ser-162 at 8% (see Fig. 3 for details) (40). The similarity of RDD spectra across the WS peak indicates absence of isomerization in that fraction. Variations in the relative abundance between the WI/WS fractions may correlate with perturbed oligomerization, as discussed in more detail below.

This approach can also be applied to more complex systems, as illustrated by the chromatograms for separation of αB-^57^APSWFDTGLSEMR^69^, from which nine different isomers were identified in the WI fraction (Fig. 2*C*, *bottom*). Synthetic standards of selected candidates were used to determine that the original all-l peptide comprises only 14% of the total abundance in the WI fraction (peak 7, which dominates the WS fraction). The ^αB^d-Ser-59 epimer is present in 5.6% abundance (peak 4) in the WI fraction and 1.4% in the WS fraction (see Fig. S3 for more information). Interestingly, when αB-^57^APSWFDTGLSEMR^69^ is phosphorylated at ^αB^Ser-59, only a single isomer is detected in the WS fraction (Fig. 2*D*), and a small number of isomers are detected in the WI fraction. This suggests that there is a relationship between phosphorylation and isomerization. Finally, the WS and WI fractions of αB-^108^QDEHGFISR^116^ both contain multiple isomers of ^αB^Asp-109 (Fig. 2*E*), but the relative abundance of ^αB^l-isoAsp-109 increases from 5.1 to 55.4% in the WI portion. This dramatic difference may suggest that isomerization of ^αB^Asp-109 influences aggregation propensity. Upon detailed examination, it is clear that isomerization and epimerization are occurring in regions known to be critical for the proper function and assembly of the α-crystallins. Furthermore, these results hint at structural consequences (as suggested previously in relation to observations of aggregation (16, 17)), but more detailed investigation is required to reveal how these modifications exert structural influence at the molecular level.

### Epimerization of the C-terminal region compromises binding to α-crystallin domain

**Figure 4.**
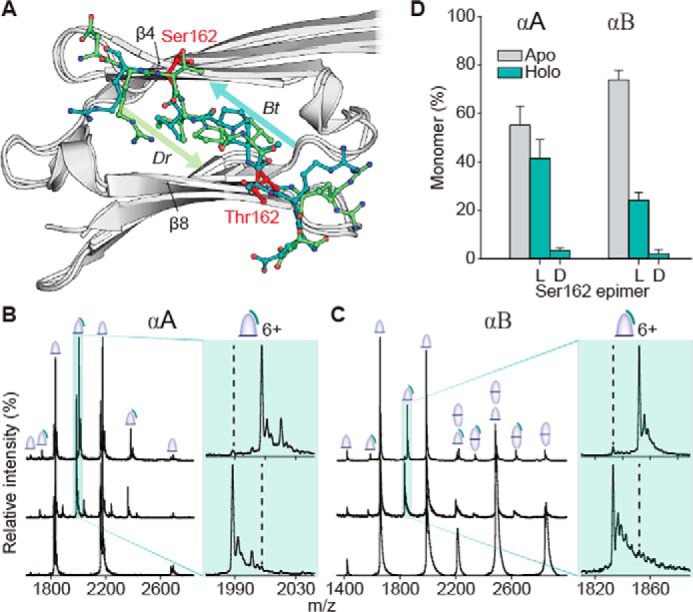
**Competition experiments reveal a strong preference for l-over d-Ser-162 binding to both αA and αB.** *A,* aligned crystal structures of the α-crystallin domain (*gray*) with C-terminal peptide bound in two alternate orientations. *Arrows* indicate orientation (N → C) of bound peptides (*green* and *teal*). Equivalent isomerization sites (Ser-162 in αA or Thr-162 in αB) are shown in *red. B,* native mass spectra of αA core alone (*bottom*) and mixed with 4:1 ERAIPVSRE and GERAIPVSREG (*middle,* S = d-Ser). As seen in the magnification of 6+ peak, *bottom spectrum*, the binding of the lighter mass l-epimer is preferred. The *upper trace* corresponds to the reverse experiment, *i.e.* ERAIPVSRE and GERAIPVSREG. *Dashed lines* guide the eye to expected positions of d-epimer–bound peaks. *C,* equivalent experiments using the core of αB yield similar results. *D,* relative average fractions of free *versus* bound monomer cores from competition experiments, using all 5+, 6+, and 7+ charge states for quantitation. *Error bars* represent 95% confidence intervals. Crystal structures are as follows: bovine, *Bt*, in *teal*, PDB 3L1F, and zebrafish, *Dr*, in *green*, PDB 3N3E.

Ser-162 from αA is an epimerized residue located adjacent to the highly conserved palindromic IX(I/V) motif within the C-terminal region that binds to a groove in the α-crystallin domain (Fig. 2*A*). Examination of the available crystal structures (Fig. 4*A*) reveals that the C-terminal tail can occupy the groove in the β8 → β4 direction or the inverse direction (β4 → β8), stabilized by hydrogen bonding between the peptide backbones in either case (41). Although the binding to this groove is dynamic and can occur through two different states, these interactions are crucial for proper oligomerization, and prior work has shown that point mutations can influence the kinetics and thermodynamics of assembly (42).

To quantify the effect of ^αB^Ser-162 epimerization on binding within this groove, we conducted a series of native MS experiments similar to previous work examining interactions between the α-crystallin core domain and peptide portions of the C-terminal tail (42). This native MS approach shows excellent agreement with NMR-based titrations (43), with the added benefit of being able to accommodate the heterogeneity of the system that other methods average over. The core domains, when analyzed alone, yield peaks corresponding to monomer (αA) and both monomer and dimer (αB) (Fig. 4, *B* and *C*, *lower spectra*). This suggests that the αA dimer interface is slightly weaker than that of αB, although the native dimer complex can be preserved and detected with native MS for both proteins. Competition binding experiments between the α-crystallin domain and palindromic peptides containing either d- or l-Ser-162 are shown in the middle and upper mass spectra of Fig. 4, *B* and *C*, and summarized in *D*. The canonical all l-amino acid form of the peptide, ERAIPVSRE, and a d-isomer variant made distinguishable by addition of glycine to both termini, GERAIPVSREG (S = d-Ser), were added at a 4:1 peptide/α-crystallin domain molar ratio. The dominant adduct observed corresponds to the binding of the canonical peptide, with the d-isomer representing only a small fraction of the binding (Fig. 4*D*).

To eliminate the possibility that the additional glycine residues might be responsible for the difference in binding, the inverse experiment was conducted with the appended-glycine l-isomer (Fig. 4, *B* and *C*, and *insets, upper spectra*). Binding of the l-isomer dominates again in both systems (Fig. 4*D*), and the data can be used for quantification (44, see Table S5) of the dissociation constants: αA core l-Ser = 48 μm, αB core l-Ser = 115 μm, αA core d-Ser = 650 μm, and αB core d-Ser = 1380 μm. Based on these values, epimerization leads to destabilization of ΔΔ*G* = ∼6 kJ/mol. All of these results are consistent with d-^αA^Ser-162 significantly inhibiting proper interaction between the C-terminal palindrome and the β4–β8 groove in the α-crystallin domain.

We have previously shown that removal of the side chain at the equivalent position in αB (^αB^T162A) leads to a weaker interaction in the peptide–α-crystallin domain system and faster subunit-exchange of full-length αB (42). The present data suggest that epimerization of ^αA^Ser-162 similarly weakens binding, which would also be expected to perturb the dynamics and chaperone activity of αA. Furthermore, because the diversity of binding modes between the C-terminal tail and the α-crystallin domain promotes polydispersity and aids in preventing crystallization (41), disruption of this binding may lead to increased oligomerization and explain the high abundance of the isomerized peptide in the WI lens fraction. Because both chaperone activity and polydispersity are presumed to help maintain lens transparency (31, 45), epimerization of ^αA^Ser-162 is a likely contributor to age-related protein aggregation within the lens.

### Phosphorylation is precluded by epimerization of ^αB^Ser-59 and affected by isomerization of ^αB^Asp-62

Many observations suggest that ^αB^Ser-59 is a structurally important site, including solid-state NMR data showing ^αB^Ser-59 is involved in various inter-monomer contacts (46). As illustrated in Fig. 2*C*, the peptide containing ^αB^Ser-59 and ^αB^Asp-62 is highly isomerized in a normal lens. In addition, the degree of epimerization at Ser-59 increases in cataractous lenses (8). Ser-59 is also a primary site of phosphorylation in αB, which helps regulate oligomeric size and activity (47). However, within the lens phosphorylation is primarily observed in the younger cortex (48).

**Figure 5.**
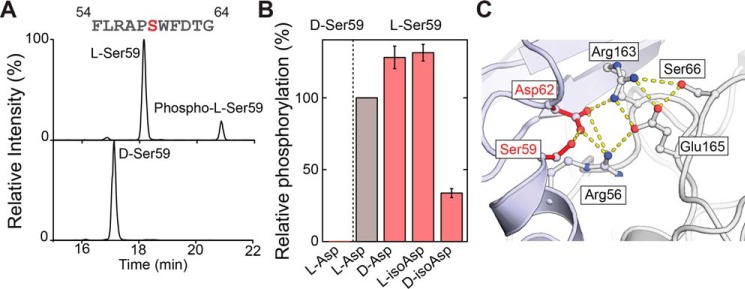
*A,* extracted ion chromatograms following incubation of FLRAPSWFDTG-NH_2_ and FLAPSWFDTG-NH_2_ (S = d-Ser) with MAPKAPK-2 reveal that d-Ser is not a viable phosphorylation substrate. *B,* relative degree of phosphorylation for Asp and Ser isomers of FLRAPSWFDTG-NH_2_. *C,* salt-bridge model (PDB 2YGD) of an N-terminal oligomeric interface involving Ser-59 and Asp-62. Hydrogen bonds are shown using *dashed yellow lines*.

To investigate the influence of ^αB^Ser-59 epimerization and ^αB^Asp-62 isomerization on phosphorylation, we incubated the corresponding synthetic isomers of FLRAPSWFDTG-NH_2_ with the native kinase, MAPKAPK-2 (49). The extracted ion chromatograms for both peptides reveal that the ratio of l-/d-Ser-59 phosphorylation is ∼240:1 after 2 h and ∼350:1 after 12 h of incubation (Fig. 5, *A* and *B*), indicating that the d-Ser-59 isomer is a poor substrate for the kinase. Interestingly, examination of the phosphorylated peptide in the lens reveals only minor isomerization (Fig. 2*D*), offering sharp contrast to the abundant isomerization of the unmodified peptide. This suggests that modifications elsewhere on the peptide may also inhibit phosphorylation, or that phosphorylation prevents isomerization, or both. Additional *in vitro* experiments confirmed that phosphorylation is inhibited by d-isoAsp-62, but slightly enhanced by d-Asp or l-isoAsp at the same position (Fig. 5*B*). In sum, the native kinase activity is affected by all three non-native Asp isomers and essentially prevented by epimerization of Ser. These results provide a potential explanation for previous experiments that failed to isolate phosphorylated d-Ser from erythrocytes (50).

Ser-59 and Asp-62 have also been suggested to be involved in an interfacial salt-bridge cluster with Arg-163 and Glu-165 of an adjacent monomer (51). In this model, all four residues are in close proximity, with isomerization of Asp-62 or Ser-59 both likely to perturb the dynamics of the salt bridge and disrupt the interface (Fig. 5*C*). Similarly, phosphorylation of Ser-59, which resides 2.4 Å (O–O distance) from Asp-62, is likely to disrupt this salt bridge network and may account for the reduced oligomer size observed for phosphorylation mimics of Ser-59 (52). Disruption of this salt-bridge cluster is explored further in experiments with recombinant proteins below.

### Isomerization of ^αB^Asp-109 breaks dimer-stabilizing salt bridge and leads to insolubility

Fig. 2*E* illustrates abundant isomerization of ^αB^Asp-109, which is known to form an inter-monomer salt bridge with ^αB^Arg-120 in the AP_II_ register (41, 46, 53), the most populated state in solution (54). Mutation at either site in the salt bridge frequently leads to malfunction and disease (53–56). For example, the R120G mutant is genetically linked to desmin-related myopathy (57), whereas mutation of ^αB^Asp-109 is associated with myofibrillar myopathy (58) and cardiomyopathy (59). To probe the structural consequences of ^αB^Asp-109 isomerization on the dimer interface of αB, *in silico* mutation and MD simulations were utilized.

**Figure 6.**
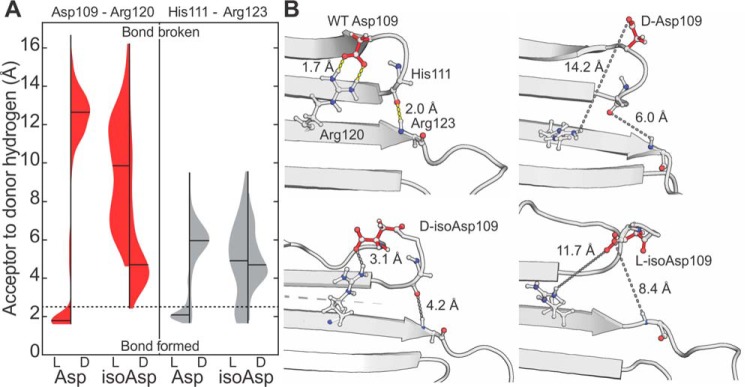
*A,* distance distributions between Asp-109 and Arg-120 (*left, red*) and His-111 and Arg-123 (*right, gray*) from MD simulations. Violin plots are shown for each isomer of Asp-109; means are marked with *black lines*, with the kernel densities normalized to have the same maximum heights. Lengths <2.5 Å can be considered to correspond to bond formation (neglecting consideration of the bond angles), whereas those longer represent absence of the bond (boundary demarcated by *dashed line*). In all isomers other than l-Asp, the hydrogen-bond donor and acceptors are located too far apart for bond formation the vast majority of the time. *B*, selected frames from MD simulations highlighting breakage of hydrogen bonds profiled in *A* and resultant interface destabilization. *Yellow dashes* indicate H-bonds; *short gray dashes* show concomitant distances following isomerization of Asp-109; *long gray dashes* mark the antiparallel dimer interface. His-111 and Arg-123 side chains have not been shown, for clarity.

The results obtained from all-atom simulations extending >150 ns are illustrated in Fig. 6. The Asp-109–Arg-120 salt bridge is stable for the l-Asp isomer, with a mean acceptor–to–donor hydrogen bond distance under 2 Å (Fig. 6*A*, *left*). For all other isomers, d-Asp-109, l-isoAsp-109, or d-isoAsp-109, the salt bridge with Arg-120 is disrupted, and the average distances increase significantly (Fig. 6*A*, *left*). To determine the influence of Asp isomerization on the stability of the dimer interface, we monitored the distances between the final backbone hydrogen bond partners (His-111 and Arg-123). Reasonable hydrogen bond distances are only maintained for the l-Asp isomer, with all other isomers producing elongated distances (Fig. 6*A*, *right*). These results are illustrated by structural snapshots in Fig. 6*B*. For the l-Asp isomer (*upper left*), a backbone hydrogen bond between His-111 and Arg-123 links together the ends of β6 + 7 strands. For the other three isomers, these partners have been shifted to noninteracting distances due to disruption of the β-sheet dimer interface. It should be emphasized that these simulations model only one of two identical salt bridges that stabilize the dimer interface. In the situation where two modified residues occupied both ends of the dimer interface, the resulting destabilization would be expected to be significantly worse. The results from these simulations offer an explanation for the observed partitioning of each isomer extracted from the lens in Fig. 2*E*. Specifically, the abundance of isomerized Asp-109 residues is much higher in the WI fraction, whereas l-Asp-109 is the virtually the only isomer present in the WS fraction, suggesting a single isomerization event at Asp-109 is sufficient to drive insolubility. The isomerization-induced loss of the dimer interface mimics the effects of the R120G mutation, which is known to cause protein aggregation (56) and increase the diversity of conformational states (60).

### Mimicking breakage of interfacial bonds by isomerization leads to aberrant oligomerization

**Figure 7.**
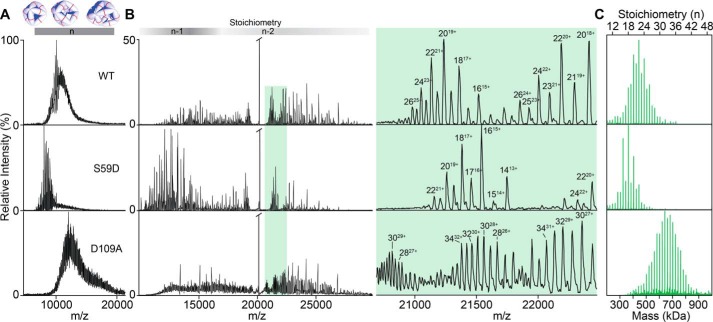
*A,* native MS of intact oligomeric assemblies of WT, S59D (phosphomimic), and D109A (isoAsp-mimic) αB, which are not directly assignable due to overlap of a multitude of charge states and stoichiometries. *B,* native MS with CID of the ions from *A. Shading above* shows regions where oligomers (*n*-mers) have lost one (*n*-1) or two (*n*-2) subunits. Detailed view of the region highlighted in *green* shows that CID resolves the charge states of the oligomers. *C,* reconstructed oligomeric distributions. Data were charge deconvolved and then corrected to account for stripped subunits.

Our examination of the amino acid environment around both Ser-59 and Asp-109 revealed that in both cases modification at these sites would be likely to impact oligomerization. Specifically, we noted that ^αB^Ser-59 appears to be part of a network of salt bridges, which is unlikely to accommodate a bulky negative charge without significant rearrangement (Fig. 5*C*, above). To test this prediction, we generated the phosphorylation mimic in which ^αB^Ser-59 was mutated to Asp (^αB^S59D) and compared it to the WT with native MS. Both proteins gave mass spectra featuring a broad region of signal at high *m*/*z*, indicative of a polydisperse ensemble of oligomers and consistent with previous spectra of αB (Fig. 7*A*) (61). Notably, the signal is at slightly lower *m*/*z* values for the phosphomimic, consistent with a shift to smaller stoichiometries. To quantify this change, we performed collisional activation to remove highly charged monomers from the parent oligomers, resulting in lower “charge-stripped” oligomers that are well resolved and can be deconvolved into an oligomeric distribution (Fig. 7, *B* and *C*) that faithfully reproduces the distribution present in solution (27, 61, 62). S59D yields a distribution centered on an 18-mer, smaller than the WT, and with a stronger preference for oligomers with an even number of subunits (Fig. 7*C*). This suggests that phosphorylation of Ser-59, which regulates activity and localization of αB (47), leads to destabilization of the larger oligomers, likely at inter-dimer interfaces involving the N-terminal region. Similarly, isomerization of Ser-59 or Asp-62 would also be expected to disrupt the salt-bridge chain in Fig. 5*C* and impact oligomerization in a comparable fashion. Generally, previous results have found that phosphorylation at Ser-59 increases chaperone activity, suggesting that larger oligomers serve to “store” chaperone capacity that can be released on demand (35, 47, 63). Isomerization at Ser-59 or Asp-62 would also interfere with this regulation mechanism.

Although the S59D mutant is an effective mimic of phosphorylated Ser-59, direct probing of the structural impacts of isomerization and epimerization is more problematic. It is not possible to use site-directed mutagenesis to insert d-residues or isoAsp into proteins, nor, given that both modifications are spontaneous, is it feasible to induce isomerization or epimerization in a site-specific and controllable fashion. Direct investigation of the influence of isomerization at Asp-109 on oligomeric assembly is therefore not feasible in the absence of whole-protein synthesis. Nevertheless, the MD results show that the major consequence of l-Asp isomerization is disruption of the salt bridge with Arg-120. This outcome can be mimicked with a D109A mutant, where the acidic partner in the salt bridge has been removed. Native MS experiments with this mimic revealed oligomeric assemblies larger than those observed with the WT protein and a lower preference for even stoichiometries (Fig. 7*C*). Complementary results were also obtained by light-scattering experiments that showed a significantly increased propensity for aggregation of D109A relative to the WT (see Fig. S4). These results are consistent with trends observed for the R120G variant, in which the same salt bridge is disrupted (30). In both cases, the dimer interface is weakened and oligomer size increases. This may explain why the l-isoAsp variant is only observed in appreciable amounts in the insoluble fraction obtained from lenses (see Fig. 2*E*). Overall, the results in Fig. 7 confirm that subtle changes that mimic isomerization can impact oligomerization toward larger or smaller sizes even in the context of the full-length proteins.

## Conclusion

Isomerization and epimerization are prevalent PTMs in long-lived proteins such as the crystallins found in the eye lens. Although these modifications are difficult to detect and cannot be probed by site-directed mutagenesis, we have demonstrated that they can cause significant structural perturbation and loss-of-function. Epimerization of a single serine residue is sufficient to inhibit noncovalent recognition needed to maintain proper interface strengths and dynamics. Furthermore, epimerization of serine or isomerization of nearby residues alters phosphorylation and any functionality derived from it. By redirecting and altering the peptide backbone, aspartic acid isomerization can also inhibit kinase recognition and disrupt native salt-bridge interactions, leading to improper oligomer assembly and size.

These observations show that what might appear to be innocuous PTMs can disrupt protein structure. It is possible to detect these modifications if they are present at 1% relative abundance to the unmodified canonical peptide (64). Given that long-lived proteins are also associated with many other diseases, including Alzheimer's and Parkinson's (1, 65, 66), it is likely that many of the structural issues highlighted herein also contribute to loss-of-function in these pathologies. The spontaneous nature of isomerization and epimerization dictates that repair will be unrealistic in most cases, suggesting that the best strategy for avoiding loss-of-function due to such modifications would be increasing protein turnover to prevent their occurrence in the first place.

## Materials and methods

### Protein expression and purification

Core αB (cABC, residues 68–153) was expressed in *E. coli* and purified as described previously (54). A gene insert encoding core αA (cAAC, residues 59–153) was purchased from Integrated DNA Technologies and inserted into a pET28a vector linearized with BamHI and XhoI (New England Biolabs) using an In-Fusion HD cloning kit (New England Biolabs) to generate a tobacco etch virus–cleavable His-tagged construct. This was expressed and purified in the same manner as cABC with addition of 5 mm BME in all buffers prior to size-exclusion chromatography, resulting in some population of BME-adducted protein visible in the spectra in Fig. 4. Core domains were stored in 100 mm NaCl, 20 mm Tris, pH 8, at −80 °C until use. Full-length αB was expressed and purified as described previously (54). Mutations S59D and D109A were introduced using a QuikChange site-directed mutagenesis kit (Agilent), and mutants were expressed and purified in the same manner as WT. Full-length proteins were stored in MS buffer (200 mm ammonium acetate, pH 6.9) at −20 °C until use. Concentrations were determined by UV absorbance at 280 nm.

### Native MS of core domains and peptides

Spectra were collected using a previously described protocol (67) on a Synapt G1 IM-QToF mass spectrometer (Waters) with parameters as follows: capillary 1.5 kV, sampling cone 40 V, extraction cone 3 V, backing pressure 3.1 mbar, trap gas (argon) 3 ml min^−1^, trap cell voltage 10 V, transfer cell voltage 8 V. Ion mobility was enabled with parameters in the mobility cell: IMS gas flow 22 ml min^−1^, IMS wave velocity 320 m s^−1^, IMS wave height 5.5 V. Proteins were buffer exchanged into 200 mm ammonium acetate, pH 6.9, using a Biospin-6 column (Bio-Rad). All spectra were recorded at 10 μm based on concentration measurement post-buffer exchange. Samples were introduced using gold-coated capillaries prepared in-house. Lyophilized peptides-Ct (ERAIPVSRE) and G-Ct-G (GERAIPVSREG) with l- or d-Ser- were resuspended in milliQ H_2_O to a stock concentration of 1 mm and then diluted in MS buffer and mixed with protein immediately prior to analysis to a final concentration of 40 μm each for competition experiments. For quantitation, monomeric species were extracted in DriftScope (Waters) and intensities recorded from MassLynx using all resolved adduct peaks in addition to *apo* for 5+, 6+, and 7+ charge states. Data are reported as the mean ± S.D. for three replicates.

### Native MS of full-length αB and mutants

Spectra were collected on a modified QExactive hybrid quadrupole-Orbitrap mass spectrometer (ThermoFisher Scientific) optimized for transmission of high-mass complexes (68). Protein concentration was 15 μm by monomer. Capillary voltage was 1.4 kV in positive ion mode with source temperature 200 °C and S-lens *RF* 200%. UHV pressure (argon) was between 1.4 × 10^−9^ and 1.7 × 10^−9^ mbar. In-source trapping fragmentation voltage ranged from −150 to −180 V. Ion transfer optics were as follows: injection flatapole 10 V, inter-flatapole lens 8 V, bent flatapole 6 V, transfer multipole 4 V, C-trap entrance lens 3 V. Nitrogen was used in the HCD cell and HCD energy was 0 V for intact spectra and tuned for optimal dissociation of each protein for CID spectra, ranging from 200 to 230 V. Resolution was kept at 17,500 at *m*/*z* = 200 for a transient time of 64 ms and the noise threshold was set to 3. For CID spectra, groupings of 30 microscans were combined to improve signal quality. Data were visualized using Xcalibur (ThermoFisher Scientific) and calibrated manually according to expected peak positions for WT αB-crystallin. Calibrated CID data were processed using UniDec software, which allowed for stoichiometric assignment and *post-hoc* correction for dissociated subunits (62).

### Molecular modeling

To study the effect of Asp-109 epimerization, we extracted structures from PDB 2WJ7, which features the αB-crystallin core dimer in the Aβ II register (thus enabling the formation of the Asp-109–Arg-120 salt bridge). Three models featuring d-Asp, d-isoAsp, and l-isoAsp at position 109 in one of the two monomers, respectively, were produced by modifying PDB 2WJ7 in Schrödinger Maestro. Simulation parameters for nonstandard amino acids were produced with Antechamber (69, 70). All atom types were assigned according to available ff14SB parameters. Calculations were performed using the Amber ff14SB force field (71) on the NAMD molecular dynamics engine (72). Structures were first solvated in a box of TIP3P water, their box charge neutralized by addition of Na^+^ ions, and the resulting systems energy-minimized with 2000 conjugate gradient steps. We then performed 0.5-ns steps in the NPT ensemble, with all protein α-carbons constrained by a harmonic potential. Langevin dynamics were used to impose a temperature of 300 K, using a damping of 1/ps. A constant pressure of 1 atm was imposed via a Langevin piston having a period of 200 fs and a decay of 50 fs. The system was then further equilibrated in the NVT ensemble for 1 ns, after which 200-ns production runs in the NPT ensemble were performed. In all simulation steps, Particle Mesh Ewald was used to treat long-range electrostatic interactions, with a cutoff distance of 12 Å for van der Waals interactions, and a 2-fs time step was implemented by restraining every covalent bond with SHAKE.

His-111–Arg-123 and Asp-109–Arg-120 distances were measured every 100 ps. For the latter, we report the shortest distance between each of the hydrogens of Arg-120 guanidinium, and oxygens of Asp-109 carboxylate.

## Author contributions

Y. A. L., M. P. C., D. L. R., M. T. D., J. L. P. B., and R. R. J. conceptualization; M. P. C., J. L. P. B., and R. R. J. funding acquisition; Y. A. L., M. P. C., D. L. R., and M. T. D. investigation and formal analysis; Y. A. L., J. L. P. B., and R. R. J. writing-original draft; J. L. P. B. and R. R. J. project administration; Y. A. L., M. P. C., D. L. R., M. T. D., J. L. P. B., and R. R. J. writing-review and editing.

## Supplementary Material

Supporting Information

## Footnotes

4 The abbreviations used are:

PTM: post-translational modification
PDB: Protein Data Bank
MD: molecular dynamics
CID: collision-induced dissociation
WI: water-insoluble
WS: water-soluble
BME: β-mercaptoethanol
RDD-MS: radical-directed dissociation-mass spectrometry.
