# Supplementary material for: Structural and functional consequences of age-related isomerization in α-crystallins
Source: J Biol Chem. 2019 Feb 25;294(19):7546–55. doi: 10.1074/jbc.RA118.007052 (PMC6514633; doi:10.1074/jbc.RA118.007052)
Supplement: Supporting Information [file supp_294_19_7546__index.html]

Structural and functional consequences of age-related isomerization in α-crystallins — Structural and functional consequences of isomerization — Structural and functional consequences of age-related isomerization in α-crystallins — Structural and functional consequences of isomerization — Supporting Information 

# Structural and functional consequences of age-related isomerization in α-crystallins

## Supporting Information

- Supporting Information (to be published online) - additional data and procedures
