# Supplementary material for: Structural and functional consequences of age-related isomerization in α-crystallins
Source: J Biol Chem. 2019 Feb 25;294(19):7546–55. doi: 10.1074/jbc.RA118.007052 (PMC6514633; doi:10.1074/jbc.RA118.007052)
Supplement: Supporting Information [file supp_RA118.007052_142437_2_supp_287531_pmzt8w.docx]

Supporting Information for:

Structural and Functional Consequences of Age-Related Isomerization in α‑Crystallins

Yana A. Lyon,^a^ Dylan L. Riggs,^a^ Miranda P. Collier,^b^

Matteo T. Degiacomi,^c^ Justin L.P. Benesch,^b^ Ryan R. Julian^a^

^a^Department of Chemistry, University of California, Riverside, 501 Big Springs Road, Riverside, CA 92521, USA; ^b^Department of Chemistry, Physical and Theoretical Chemistry Laboratory, University of Oxford, South Parks Road, Oxford OX1 3QZ, UK; ^c^Department of Chemistry, Durham University, South Road, Durham DH1 3LE, UK

Corresponding Authors: ryan.julian@ucr.edu, justin.benesch@chem.ox.ac.uk


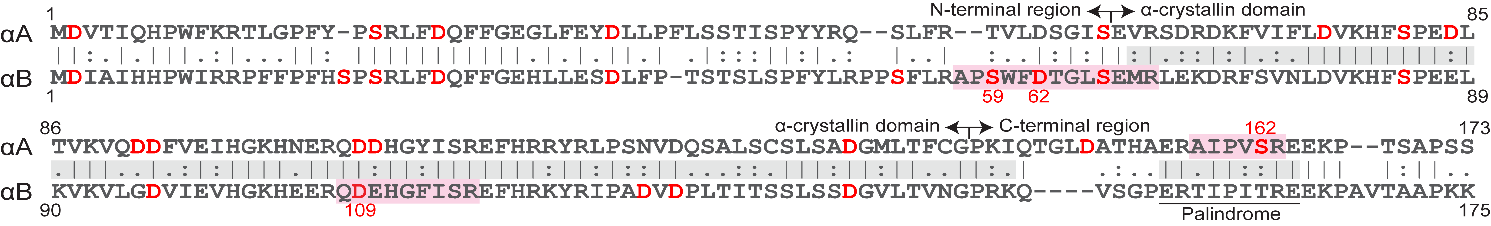


**Fig. S1.** Sequence alignment of human αA and αB with designated boundaries between domains. Red residues indicate Asp/Ser sites within peptides from 72-year-old human lens identified in numerous isomeric forms by RDD-MS. Pink regions indicate isomer-containing peptides mapping to oligomeric interfacial regions


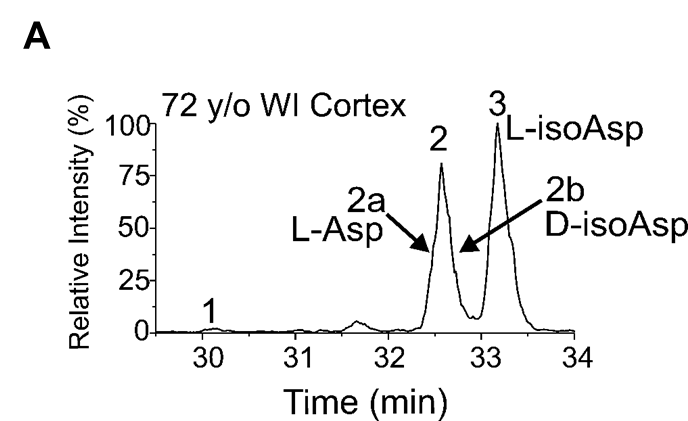

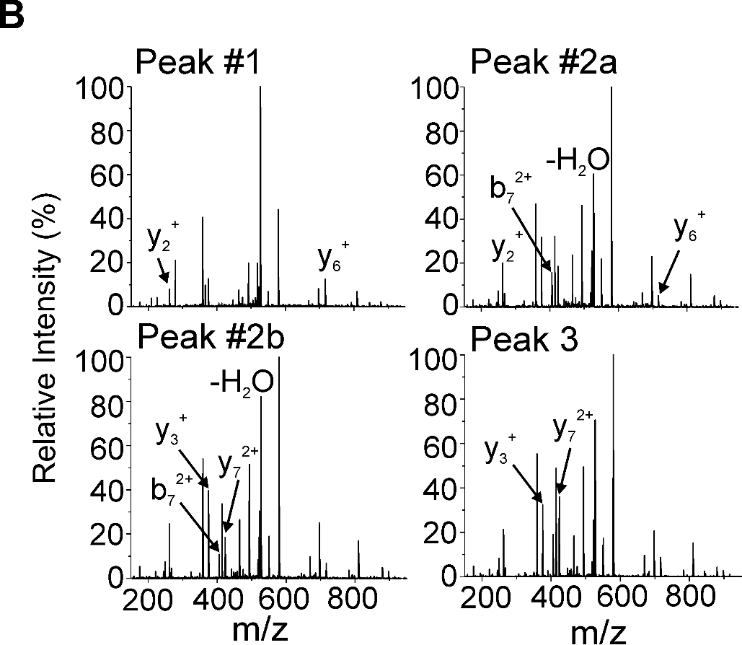
**
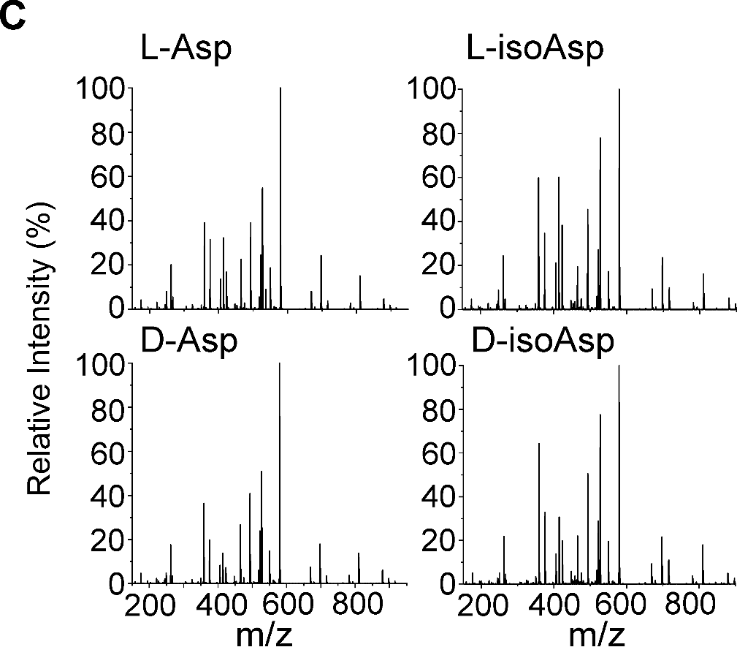
**

**Fig. S2. (**A) LC chromatogram of the ^108^pQDEHGFISR^116^ isomers from a 72 y/o WI cortex digest.(B) Four spectra resulting from collision-induced dissociation from three isomer peaks in the LC chromatogram. Peak 2 contains two co-eluting isomers and are labeled “2a and 2b”. Comparison of the fragmentation intensities in peak #1 and peak #2 show differences in the y_6_^+^ ion relative to the y_2_^+^ ion. An R_isomer_ score can be calculated using these peaks. In this example:

$$R_{\mathrm{isomer}}= \frac{R_{Peak 1}}{R_{Peak 2a}} = \frac{\frac{I_{A}}{I_{B}}}{\frac{I_{A}}{I_{B}}} = \frac{\frac{12.48}{5.13}}{\frac{7.95}{20.04}}=6.1;$$

Where I_A_ = Relative intensity of y_6_^+^, I_B_ = Relative Intensity of y_2_^+^.

This calculation is then used to compare the leading edge of peak 2 (2a) to the trailing edge of peak 2 (2b), and then peak 2b to peak 3. The R_isomer_ scores for each of these subsequent calculations are all above the threshold of 1.9, indicating that they are different isomers. The observed m/z values are as follows: y2+ (262.17), y3+ (375.33), y6+ (716.50), y7 2+ (423.25), b7 2+ (810.33), [M+2H]2+ – H2O (527.33). (C) Synthetic standards of the pQDEHGFISR are then used to identify the specific isomer in each of the peaks. This is done by comparing the fragmentation spectrum in each of the peaks to the fragmentation spectra from the authentic L-Asp, L-isoAsp, D-Asp and D-isoAsp synthetic versions. An R_isomer_ score below the 1.9 threshold allows for confident confirmation of the isomer.

**Table S1.** Idenfication of Asp isomers from ^108^pQDEHGFISR^116^ from αB in WI Cortex of 72 y/o Lens using R_isomer_ scores

| **Peak Observed from Lens** | **Synthetic standard** | **R_isomer_** |
| --- | --- | --- |
| Peak 1 | L-Asp | 5.4 |
| Peak 1 | L-isoAsp | 3.9 |
| Peak 1 | D-Asp | 5.9 |
| Peak 1 | D-isoAsp | 4.1 |

| **Peak Observed from Lens** | **Synthetic standard** | **R_isomer_** |
| --- | --- | --- |
| **Peak 2a** | **L-Asp** | **1.2** |
| Peak 2a | L-isoAsp | 2.6 |
| Peak 2a | D-Asp | 2.3 |
| Peak 2a | D-isoAsp | 2.4 |

| **Peak Observed from Lens** | **Synthetic standard** | **R_isomer_** |
| --- | --- | --- |
| Peak 2b | L-Asp | 2.7 |
| Peak 2b | L-isoAsp | 3.8 |
| Peak 2b | D-Asp | 2.4 |
| **Peak 2b** | **D-isoAsp** | **1.5** |

| **Peak Observed from Lens** | **Synthetic standard** | **R_isomer_** |
| --- | --- | --- |
| Peak 3 | L-Asp | 2.8 |
| **Peak 3** | **L-isoAsp** | **1.2** |
| Peak 3 | D-Asp | 5.1 |
| Peak 3 | D-isoAsp | 2.3 |

**Table S1** lists the results of the isomer identification of the pQDEHGFISR peaks from the WI cortex of the 72 y/o lens, and the best matching isomer is indicated in red. When matching observed peptides to synthetic standards, low R_isomer_ values are desired to indicate similarity (the opposite of what is done to distinguish isomers from each other, where high R_isomer_ values are meaningful). Peak 1 (Figure S1a) centered at 30 min is an isomer of this peptide, but does not match any of the Asp synthetics suggesting that it contains a different site of isomerization.


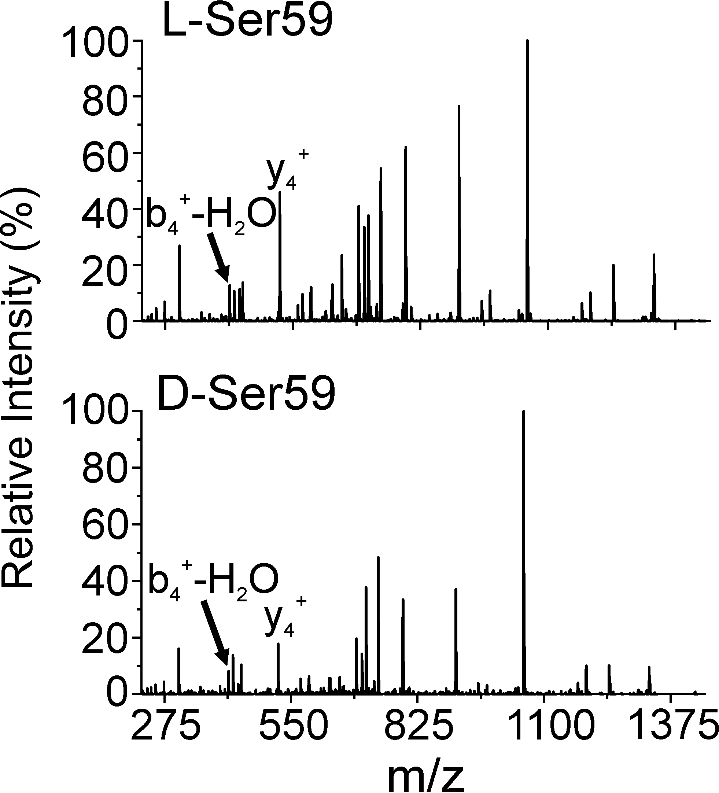


**Fig. S3.** Resulting CID spectra of the APSWFDTGLSEMR L-Ser59 and D-Ser59 synthetic standards. Comparison of the b_4_^+^ -H2O and y_4_^+^ ions yields an R_isomer_ score of 4.1 indicating that CID can be used to detect D-Ser59 in this peptide. The fragmentation patterns of these two synthetics are then checked against each of the 9 peaks, allowing for identification of both L-Ser59 and D-Ser59.

**Table S2.** Identification of L-Ser59 of ^57^APSWFDTGLSEMR^69^ from αB in WI Cortex

| **Synthetic standard** | **Peak Observed from Lens** | **R_isomer_** |
| --- | --- | --- |
| L-Ser59 | Peak 1 | 5.7 |
| L-Ser59 | Peak 2 | 3.4 |
| L-Ser59 | Peak 3 | 3.8 |
| L-Ser59 | Peak 4 | 4.2 |
| L-Ser59 | Peak 5 | 2.5 |
| L-Ser59 | Peak 6 | 8.3 |
| **L-Ser59** | **Peak 7** | **1.3** |
| L-Ser59 | Peak 8 | 2.9 |
| L-Ser59 | Peak 9 | 3.5 |

**Table S3.** Identification of D-Ser59 of ^57^APSWFDTGLSEMR^69^ from αB in WI Cortex

| **Synthetic standard** | **Peak Observed from Lens** | **R_isomer_** |
| --- | --- | --- |
| D-Ser59 | Peak 1 | 5.4 |
| D-Ser59 | Peak 2 | 4.8 |
| D-Ser59 | Peak 3 | 4.9 |
| **D-Ser59** | **Peak 4** | **1.4** |
| D-Ser59 | Peak 5 | 3.3 |
| D-Ser59 | Peak 6 | 7.6 |
| D-Ser59 | Peak 7 | 4.9 |
| D-Ser59 | Peak 8 | 4.7 |
| D-Ser59 | Peak 9 | 4.5 |

**Table S4.** Identification of Ser epimers from 4IB-158AIPVSR163 from αA in WI Cortex of 72 y/o Lens using Risomer scores

| **Peak Observed from Lens** | **Synthetic standard** | **R_isomer_** |
| --- | --- | --- |
| RT 68.42 | L-Ser | 4.6 |
| **RT 68.42** | **D-Ser** | **1.7** |

| **Peak Observed from Lens** | **Synthetic standard** | **R_isomer_** |
| --- | --- | --- |
| **RT 68.42** | **L-Ser** | **1.3** |
| RT 68.42 | D-Ser | 6.0 |


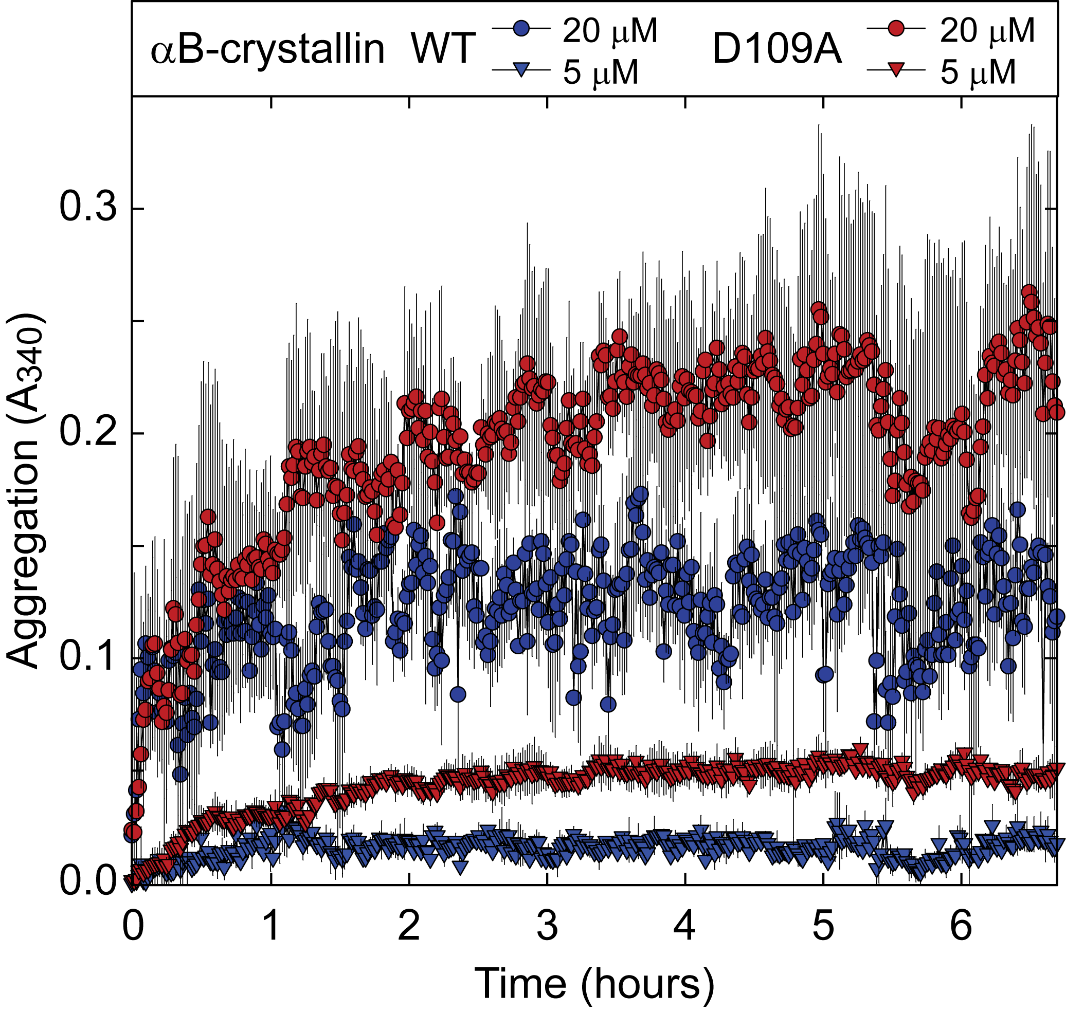


**Fig. S4.** Aggregation of αB-crystallin (WT or D109A) over time at 42°C monitored by light scattering at 340 nm. Molar concentrations correspond to monomers. Error bars represent one s.d. (n = 3). The D109A substitution predisposes αB-crystallin to form large aggregates, in agreement with the native-MS results.

**Table S5.** Calculation of approximate dissociation constants for epimer peptide binding to α-crystallin core domains.^[[1]](#endnote-1)^

|  | cAAC | cABC |
| --- | --- | --- |
| [Core]_0_ (M) | 1 × 10^-5^ | 1 × 10^-5^ |
| [L-peptide]_0_ (M) | 4 × 10^-5^ | 4 × 10^-5^ |
| [D-peptide]_0_ (M) | 4 × 10^-5^ | 4 × 10^-5^ |
| [Core]_eq_^a^ | 55.14 | 73.82 |
| [Core-L-peptide]_eq_^a^ | 3.30 | 2.12 |
| [Core-D-peptide]_eq_^a^ | 41.40 | 24.06 |
| R^b,c^ | 0.751 (0.059) | 0.326 (0.029) |
| K_D_^c,d^ | 4.8 × 10^-5^ (6.6 × 10^-4^) | 1.2 × 10^-4^ (1.4 × 10^-3^) |

^a^ Relative percentages which are proportional to concentrations, calculated from native MS binding experiments.

^b^ Abundance ratio, [Core-peptide]_eq_/[Core]_eq_

^c^ Values in parentheses correspond to D-peptide.

^d^ $\frac{{Peptide}_{0}-\frac{R_{1}}{1+R_{1}+R_{2}}{[Core]}_{0}}{R_{1}}$ where *R_1_* is the abundance ratio between the core and the peptide of interest, and *R_2_* is the ratio using the competing peptide. *(The inverse of Eq. 3 describing the K_A_ in the reference 1.)*

**Detailed Materials and Methods**

*Lens samples—*Human lenses were acquired from the National Disease Research Interchange (NDRI) (Philadelphia, Pennsylvania). The nuclei and cortices of the thawed lenses were separated using a 4mm trephine. The endcaps of each nucleus were removed by gently scraping off the outer tissue until only the dense nuclear portion remained. The nucleus and cortex were then separately homogenized in 600 μL of 50 mM Tris-HCl pH = 7.8. The lens fractions were then centrifuged at 15,100g for 20 min at 4ºC to separate the supernatant from the precipitate.

*WS fraction.* The WS supernatant was purified by dialysis against water and lyophilized. 50 μg of lyophilized powder was added to 20 μL of 50 mM NH_4_HCO_3_ buffer, pH = 7.8. Disulfide bonds were then reduced with 1.5 μL of 100 mM of dithiothreitol (DTT) at 95°C for 5 minutes. After returning to room temperature, the solution was treated with 3 μL of 100 mM iodoacetamide (IAA) and incubated at room temperature in the dark for 20 minutes.

*WI fraction.* The WI precipitate was solubilized in 6 M urea in 50 mM Tris-HCl pH=7.8 and purified by dialysis. Approximately 100 μg of the dialyzed protein (as determined by concentration based on absorbance at 280nm) was diluted to a total volume of 100 μL in 6 M urea in 50 mM Tris-HCl, pH = 8.0. Disulfide bonds were reduced with 5 μL of 200 mM DTT at 37°C for 20 minutes and then capped with 20 μL of 200 mM of IAA for one hour. The urea concentration was diluted to < 0.6 M using 50 mM Tris-HCl, 1 mM CaCl2, pH = 7.6.

Proteins were digested with trypsin for 12-16 hours at 37°C using a 50:1 protein to enzyme ratio. For samples requiring the iodobenzoate modification (i.e. RDD experiments), the digested peptides were desalted and cleaned using a peptide trap (Michrom Bioresource Inc). Approximately 5 nmoles of the digestion mixture, 10-100x excess of 4-iodobenzoic acid NHS-activated ester in dioxane and borate buffer (pH = 8.6) were combined and incubated for 1 hour at 37°C. Important: Dimethyl sulfoxide should not be substituted for dioxane in this step because it can cause aspartic acid isomerization. The modification side products at arginine and tyrosine side chains were removed by incubating the reaction mixture in 1 M hydroxylamine, pH = 8.5. The same procedure was used to modify synthetic peptide standards. These procedures have been determined previously not to yield any detectable isomerization in control experiments.^[[2]](#endnote-2)^

*Peptide and Radical Precursor Synthesis***--** All synthetic peptides were synthesized manually using standard FMOC SPPS procedures with Wang resins, or Rink Amide resins as the solid support.^[[3]](#endnote-3)^ N-hydroxysuccinimide (NHS) activated iodobenzoyl esters were synthesized by following a previous procedure. Synthetic model peptides were purified using a Jupiter Proteo column (250 mm x 4.6 mm, 4 μm, 90 Å, C12) (Phenomenex, Torrence, CA) and then lyophilized. These peptides were used to authenticate the identities of all peptide isomers and to generate calibration curves for accurate quantitation when full separation by LC was not achieved.

*Phosphorylation Assays***—**Mitogen-activated protein kinase-activated protein kinase 2 (MAPKAPK-2, also MK2) was purchased from Genetex (Irvine, CA). The kinase substrate peptides were synthesized with Rink Amide resins to prevent a charged C-terminus from interfering with kinase recognition. 100µM of each purified synthetic peptide was assayed in 25 mM HEPES buffer at pH 7.5 with 25mM MgCl_2_, and 2mM ATP in a 37 ºC sand bath. The assays were initiated by addition of 1µg kinase (for L-Ser and D-Ser assays) or 0.5µg kinase for (L-Asp, D-Asp, L-isoAsp, and D-isoAsp assays). Aliquots were taken at defined timepoints and the reaction was quenched by the addition of 1% TFA (v/v) before rapid freezing in liquid nitrogen. Samples were stored frozen until LCMS analysis. As a control, the all L- form of the FLRAPSWFDTG-NH_2_ peptide was assayed without the addition of kinase to eliminate the possibility of autophosphorylation.

*LC-MS Data Acquisition and Analysis***--** An Agilent 1100 series HPLC system (Agilent, Santa Clara, CA) with a BetaBasic column (150 mm x 2.1 mm, 3 μm, 150 Å, C18) was coupled to an LTQ mass spectrometer. Peptides were separated using a 0.1% formic acid in water (mobile phase A) and a 0.1% formic acid in acetonitrile (mobile phase B) binary system at a flow rate of 0.2 mL/min. The digestion mixtures were loaded onto the column and separated using the following gradient: 5% B to 20% B over 60 minutes, 20% B to 30% B over the next 45 minutes, 30% B to 50% B over the next 15 minutes and 50% B to 95% B over the final 10 minutes. The LTQ was operated in data-dependent mode using the Xcalibur program (Thermo Fisher Scientific). Specifically, in the CID-only LC-MS run, the first scan event was a full MS from m/z 400-2000 Da, followed by an ultrazoom and then CID. In the RDD LC-MS experiments, the laser pulses were triggered during the MS^2^ step and CID was performed as a pseudo-MS^3^ step. Due to the high photodissociation yield of the 4-iodobenzoic acid chromophore, the major peak during this step is the loss of iodine, and it is the subsequent precursor for MS^3^. The exclusion time was set to 60 seconds for the identification of peptides. For isomer identification, an inclusion mass list was added and the exclusion time was reduced to 16 seconds to enable repeated analysis of isomers.

*R_isomer_ Calculations***--** R_isomer_ represents the ratios of the relative intensities of a pair of fragments that varies the most between two isomers (R_A_/R_B_). Following acquisition of a tandem mass spectrum, R_isomer_ values are calculated for all pairs of peaks to reveal fragments that yield the best differentiation. If R_isomer_ = 1 then the two MS^n^ spectra are indistinguishable, and the species are likely not isomers. If R_isomer_ >1, a larger number indicates a higher probability that the two species are isomers. To confidently identify each of these isomers by MS^n^, we use a threshold that was determined by performing a *t*-test on the R_isomer_ values obtained by performing CID and RDD on a mixture of synthetic peptides separated by LCMS. Using 99% confidence intervals, the R_isomer_ threshold for CID is >1.9 and for RDD it is >2.4.2

*Protein expression and purification –* Core αB (cABC, residues 68-153) was expressed in *E. coli* and purified as described previously.^[[4]](#endnote-4)^ A gene insert encoding core αA (cAAC, residues 59-153) was purchased from Integrated DNA Technologies and inserted into a pET28a vector linearized with BamHI and XhoI (New England Biolabs) using an In-Fusion HD Cloning Kit (New England Biolabs) to generate a TEV-cleavable His-tagged construct. This was expressed and purified in the same manner as cABC with addition of 5 mM BME in all buffers prior to SEC, resulting in some population of BME-adducted protein visible in the spectra in Figure 3. Core domains were stored in 100 mM NaCl, 20 mM Tris, pH 8 at -80°C until use. Full length αB was expressed and purified as described previously.2 Mutations S59D and D109A were introduced using a Quik-Change Site-Directed Mutagenesis kit (Agilent) and mutants were expressed and purified in the same manner as WT. Full length proteins were stored in MS buffer (200 mM ammonium acetate pH 6.9) at -20°C until use. Concentrations were determined by UV absorbance at 280 nm.

*Native MS of core domains and peptides--* Peptides were resuspended to 1 mM in water, then diluted without additional purification. Filtration was avoided as this adds uncertainty to the concentrations. Spectra were collected using a previously described protocol^[[5]](#endnote-5)^ on a Synapt G1 IM-QToF mass spectrometer (Waters) with parameters as follows: capillary 1.5 kV, sampling cone 40 V, extraction cone 3 V, backing pressure 3.1 mbar, trap gas (argon) 3 mL min^-1^, trap cell voltage 10 V, transfer cell voltage 8 V. Ion mobility was enabled with parameters in the mobility cell: IMS gas flow 22 mL min^-1^, IMS wave velocity 320 m s^-1^, IMS wave height 5.5 V. Proteins were buffer exchanged into 200 mM ammonium acetate pH 6.9 using a Biospin-6 column (BioRad). All spectra were recorded at 10 μM based on concentration measurement post- buffer exchange. Samples were introduced using gold-coated capillaries prepared in-house. Lyophilized peptides -- Ct (ERAIPVSRE) and G-Ct-G (GERAIPVSRE) with L or D-Ser – were resuspended in milliQ H_2_O to a stock concentration of 1 mM and then diluted in MS buffer and mixed with protein immediately prior to analysis to a final concentration of 40 μM each for competition experiments. For quantitation, monomeric species were extracted in DriftScope (Waters) and intensities recorded from MassLynx using all resolved adduct peaks in addition to apo for 5+, 6+ and 7+ charge states. Data are reported as the mean +/- SD for three replicates.

*Native MS of full length αB and mutants--* Spectra were collected on a modified QExactive hybrid quadrupole-Orbitrap mass spectrometer (ThermoFisher Scientific) optimized for transmission of high-mass complexes.^[[6]](#endnote-6)^ Protein concentration was 15 μM by monomer. Capillary voltage was 1.4 kV in positive ion mode with source temperature 200°C and S-lens RF 200%. UHV pressure (argon) was between 1.4 x 10^-9^ and 1.7 x 10^-9^ mbar. In-source trapping fragmentation voltage ranged from -150 to -180 V. Ion transfer optics were as follows: injection flatapole 10 V, inter-flatapole lens 8 V, bent flatapole 6 V, transfer multipole 4 V, C-trap entrance lens 3 V. Nitrogen was used in the HCD cell and HCD energy was 0 V for intact spectra and tuned for optimal dissociation of each protein for CID spectra, ranging from 200 to 230 V. Resolution was kept at 17,500 at m/z = 200 for a transient time of 64 ms and the noise threshold was set to 3. For CID spectra, groupings of 30 microscans were combined to improve signal quality. Data were visualized using Xcalibur (ThermoFisher Scientific) and calibrated manually according to expected peak positions for WT αB-crystallin. Calibrated CID data were processed using UniDec software^[[7]](#endnote-7)^ which allowed for stoichiometric assignment and post-hoc correction for dissociated subunits.

*Molecular modeling*-- PDB 4M5T, featuring cABC (residues 67-151) with each subunit complexed with a C-terminal peptide (156-164), was exploited to assess the interactions between C-terminal peptide and crystallin domains. Two molecular dynamics simulations were prepared, one with C-terminal peptides bound as shown in the crystal structure, i.e. running from the β8 to the β4 strands of cABC, and one with the peptide bound in the opposite direction, i.e. β4 to β8. In order to build a model of the β4-β8 peptide, we selected only the alpha carbons of β8-β4 peptide, renumbered them in the opposite direction, and used them as template to align a peptide extracted from the crystal structure.

Structures were then simulated using the Amber ff14SB force field^[[8]](#endnote-8)^ on the NAMD molecular dynamics engine^[[9]](#endnote-9)^ Structures were first solvated in a box of TIP3P water, their box charge neutralized by addition of Na+ ions, and the resulting systems energy minimized with 2000 conjugate gradient steps. We then performed 0.5 ns steps in the NPT ensemble, with all protein’s alpha carbons constrained by a harmonic potential. Langevin dynamics were used to impose a temperature of 300 K, using a damping of 1 /ps. A constant pressure of 1 Atm was imposed via a Langevin piston having a period of 200 fs, and a decay of 50 fs. The system was then further equilibrated in the NVT ensemble for 1 ns, after which 200 ns production runs in the NPT ensemble were performed. In all simulation steps, Particle Mesh Ewald was used to treat long range electrostatic interactions, a cutoff distance of 12 Å was set on van der Waals interactions, and a 2 fs time step was exploited by restraining every covalent bond with SHAKE.

From each simulation, one frame per nanosecond was extracted from the production run for analysis. From each of these, hydrogen bonds between each residue part of a peptide and ones part of the crystallin domain were identified using VMD.^[[10]](#endnote-10)^ We counted the occurrences of each bond in order to determine the percentage of time two residues spend interacting. A bond was considered established if the distance between donor and acceptor atoms was less than 3 Å, and the acceptor-donor-hydrogen angle less than 20 degrees. Residue pairs forming simultaneously more than one H-bond in the same frame would be counted as bonded only once.

In order to study the effect of Asp109 epimerization we exploited PDB 2WJ7, featuring a cABC dimer in Aβ II register (thus enabling the formation of the Asp109-Arg120 salt bridge). Three models featuring D-Asp, D-isoAsp and L-isoAsp at position 109 in one of the two monomers, respectively, were produced by modifying PDB 2WJ7 in Schrödinger Maestro. Simulation parameters for non-standard amino acids were produced with Antechamber^[[11]](#endnote-11),^^[[12]](#endnote-12)^ All atom types could be assigned according to available ff14SB parameters. All models were simulated in explicit solvent for 150 ns following the same simulation protocol described above. His111-Arg123 and Asp109-Arg120 distances were measured every 100 ps. For the latter, we report the shortest distance between each of the hydrogens of Arg120 guanidinium, and oxygens of Asp109 carboxylate. The percentage of time a hydrogen bond is established was determined as described above, and averaged over the three simulations

1. El-Hawiet, A., Kitova, E. N., Liu, L., and Klassen, J. S. (2010) Quantifying Labile Protein–Ligand Interactions Using Electrospray Ionization Mass Spectrometry. *J. Am. Soc. Mass Spectrom.* **21**, 1893–1899. [↑](#endnote-ref-1)
2. Tao Y, Julian RR (2014) Identification of amino acid epimerization and isomerization in crystallin proteins by tandem LC-MS. *Anal Chem* 86(19):9733–9741. [↑](#endnote-ref-2)
3. Hood, C. A., Fuentes, G., Patel, H., Page, K., Menakuru, M., Park, J. H. (2008) Fast Conventional Fmoc Solid-Phase Peptide Synthesis with HCTU, *J. Pept. Sci.* *14*, 97–101. [↑](#endnote-ref-3)
4. Hochberg GKA, et al. (2014) The structured core domain of B-crystallin can prevent amyloid fibrillation and associated toxicity. *Proc Natl Acad Sci* 111(16):E1562–E1570. [↑](#endnote-ref-4)
5. Kondrat FDL, Struwe WB, Benesch JLP (2014) Native mass spectrometry: Towards high-throughput structural proteomics. *Structural Proteomics: High-Throughput Methods: Second Edition*, pp 349–371. [↑](#endnote-ref-5)
6. Rose RJ, Damoc E, Denisov E, Makarov A, Heck AJR (2012) High-sensitivity Orbitrap mass analysis of intact macromolecular assemblies. *Nat Methods* 9(11):1084–1086. [↑](#endnote-ref-6)
7. Marty MT, et al. (2015) Bayesian Deconvolution of Mass and Ion Mobility Spectra: From Binary Interactions to Polydisperse Ensembles. *Anal Chem* 87(8):4370–4376. [↑](#endnote-ref-7)
8. Maier JA, et al. (2015) ff14SB: Improving the Accuracy of Protein Side Chain and Backbone Parameters from ff99SB. *J Chem Theory Comput* 11(8):3696–3713. [↑](#endnote-ref-8)
9. Phillips JC, et al. (2005) Scalable molecular dynamics with NAMD. *J Comput Chem* 26(16):1781–1802. [↑](#endnote-ref-9)
10. Humphrey W, Dalke A, and Schulten K (1996) VMD: Visual molecular dynamics. *J Mol Graph* 14, 33-38. [↑](#endnote-ref-10)
11. Wang J, Wang W, Kollman PA, Case DA (2006) Automatic atom type and bond type perception in molecular mechanical calculations. *J Mol Graph Model* 25, 247–260. [↑](#endnote-ref-11)
12. Wang J, Wolf RM, Caldwell JW, Kollman PA, Case DA (2004) Development and testing of a general amber force field. *J Comput Chem* 25(9):1157–1174. [↑](#endnote-ref-12)
